# Social isolation impairs the persistence of social recognition memory by disturbing the glutamatergic tonus and the olfactory bulb-dorsal hippocampus coupling

**DOI:** 10.1038/s41598-018-36871-6

**Published:** 2019-01-24

**Authors:** Ana F. Almeida-Santos, Vinícius R. Carvalho, Laura F. Jaimes, Caio M. de Castro, Hyorrana P. Pinto, Tadeu P. D. Oliveira, Luciene B. Vieira, Márcio F. D. Moraes, Grace S. Pereira

**Affiliations:** 1 Núcleo de Neurociências, Departamento de Fisiologia e Biofísica, Instituto de Ciências Biológicas, Universidade Federal de Minas Gerais, Belo Horizonte, Brazil; 2 Programa de Pós-graduação em Engenharia Elétrica, Universidade Federal de Minas Gerais, Belo Horizonte, Brazil; 3 Laboratório de Neurofarmacologia, Departamento de Farmacologia, Instituto de Ciências Biológicas, Universidade Federal de Minas Gerais, Belo Horizonte, Brazil

## Abstract

The absence of companion may jeopardize mental health in social animals. Here, we tested the hypothesis that social isolation impairs social recognition memory by altering the excitability and the dialog between the olfactory bulb (OB) and the dorsal hippocampus (dHIP). Adult male Swiss mice were kept grouped (GH) or isolated (SI) for 7 days. Social memory (LTM) was evaluated using social recognition test. SI increased glutamate release in the OB, while decreased in the dHIP. Blocking AMPA and NMDA receptors into the OB or activating AMPA into the dHIP rescued LTM in SI mice, suggesting a cause-effect relationship between glutamate levels and LTM impairment. Additionally, during memory retrieval, phase-amplitude coupling between OB and dHIP decreased in SI mice. Our results indicate that SI impaired the glutamatergic signaling and the normal communication between OB and HIP, compromising the persistence of social memory.

## Introduction

An ever-growing body of evidence has been showing that social isolation (SI) in general, and perceived social isolation (loneliness) in particular, have significant negative effects on human health^1–4^. There is also anatomical evidence suggesting that white matter density in areas related to social cognition is negatively correlated with Loneliness Scale responses in adults^5^. Additionally, loneliness might mediate the relationship between the amygdala volume and social distress scores^6^.

Rodents are also sensitive to SI and, as socially innate animals, prefer social housing, instead of isolation^7^. Particularly the post-winning SI induces long-lasting effects on cerebral function^8–10^, emotional behaviors^11–13^ and learning and memory^14,15^. On contrary, much less is known about the mechanisms underlying SI effects on adult animals. A brief period of SI, such as 24 h, is enough to impair social memory^16^ and to cause synaptic changes in dopaminergic neurons from the dorsal raphe nucleus^17^. Interestingly, activating those dopaminergic neurons increased social preference in mice^17^. Longer periods of SI, such as one week, impaired social memory persistence and olfaction, without compromising other hippocampus-dependent memories^18,19^.

Regarded as the first relay in the olfactory neural pathway, the olfactory bulb (OB) holds functional units, known as glomeruli, where the olfactory sensory neurons synapse with mitral cells. Mitral and tufted cells direct their glutamatergic projections to a wide number of brain regions^20^, such as the dentate gyrus of the hippocampus (HIP) via the lateral entorhinal cortex and the lateral perforant pathway^21^. In turn, part of the top-down modulation of the OB originates from the olfactory and limbic system and are essentially glutamatergic^22^. Mitral/tufted cells dendrites can also excite each other via glutamate spillover^23^. Therefore, any disturbance in the glutamatergic signaling would compromise the OB function. For instance, tufted and periglomerular cells from mice that underwent to olfactory deprivation presented higher amplitude of excitatory postsynaptic current (mEPSCs), mediated by the glutamate receptor AMPA^24^.

Social memory can be defined as the ability to recognize conspecifics^25,26^ and may be considered a hippocampus-dependent memory^16,27–29^. Furthermore, social memory processing relies on olfactory cues^30–32^ and protein synthesis in the OB^27^. However, it is yet to be determined whether OB and hippocampus interact to modulate social memory, although the relationship between those neural substrates had been already observed during a discriminative olfactory task^33^.

Here, we tested the hypothesis that social isolation alters the excitability and the dialog between the OB and HIP, limiting the duration of social memory. As an indicative for excitability, we evaluated the glutamate release in synaptosomes from OB and HIP. We also tested whether glutamate levels were driving the social memory consolidation by site-target injections of specific agonists and antagonist of AMPA and NMDA receptors. In addition, we evaluated whether the SI would also disturb the neural circuits connecting the OB and the HIP during social memory recollection. Our results show that SI changed the OB and HIP glutamatergic tonus, in opposite direction, causing a social memory deficit. We suggest that disturbing glutamatergic signaling compromised the normal communication between OB and HIP, impairing social memory persistence.

## Methods

### Subjects

Adult (8–12 weeks old) male Swiss mice were kept in groups of 4–5 animals (group-housed, GH) or alone (social isolated, SI) during 7 days, in a standard plastic cage (28 × 17 × 12 cm). Juveniles (21 days old) of the same strain and sex were kept in a standard plastic cage (28 × 17 × 12 cm). All animals were maintained in a climate-controlled environment (22 ± 2 °C, humidity at 55 ± 10%) under a 12 h dark- 12 h light cycle. All behavioral experiments were performed during the light phase. Both food and water were available *ad libitum*.

All experimental procedures were approved by The Animal Use Ethics Committee of Universidade Federal de Minas Gerais (CEUA), under the protocol number 55/2015. All experiments were performed in accordance with the National Institutes of Health guide for the care and use of Laboratory animals (NIH Publications No. 8023, revised 1978).

### Synaptosomes isolation

Animals were decapitated and had their hippocampus and olfactory bulb removed and homogenized in 1:10 (w/v) 0.32 M sucrose solution containing dithiothreitol (0.25 mM; MOLECULAR PROBES) and EDTA (1 mM; SIGMA-ALDRICH). Homogenates were submitted to low-speed centrifugation (1000 × g × 10 min) and synaptosomes were purified from the supernatant by discontinuous Percoll-density gradient centrifugation [SIGMA-ALDRICH^34^]. The isolated nerve terminals were re-suspended in Krebs–Ringer–HEPES solution (KRH; 124 mM NaCl, 4 mM KCl, 1.2 mM MgSO_4_, 10 mM glucose, 25 mM HEPES, pH 7.4) without adding CaCl_2_, to a concentration of approximately 10 mg/mL. For measurement of glutamate release, aliquots of 30 μl were prepared and kept on ice until use.

### Measurement of continuous glutamate release

Glutamate release was measured by the increase of fluorescence due to the production of NADPH in the presence of type II glutamate dehydrogenase (SIGMA-ALDRICH) and NADP+^35^. This assay is based on the reaction involving GDH (glutamate dehydrogenase), NADP+ (nicotinamide adenine dinucleotide phosphate), NADPH and glutamate. When glutamate is released by synaptosomes it undergoes oxidation by the enzyme GDH and NADP+ is the acceptor of the electron oxidation. The NADPH being excited by light at a wavelength of 360 emits light at the wavelength of 450 nm, which is detected by a photomultiplier in the spectrofluorimeter. Thus, the glutamate released by the synaptosomes is quantified^35^. Since the reaction can occur both for the formation of α-Ketoglutarate or L-glutamate, an excess of NADP+ favors the direction of the reaction for the formation of α-ketoglutarate.

The procedure was performed as follows: the reaction medium containing a mixture of synaptosomes (approximately 30 μg of protein/well), CaCl_2_ (1 mM) and NADP+ (1 mM; SIGMA-ALDRICH) in KRH were transferred to Elisa microplates (300 μl/well) attached to a spectrofluorimeter (SYNERGY 2). After 3 min, glutamate dehydrogenase (35 units per well) was added and the reading was restarted until the fluorescence reached balance (approximately 10 min). Synaptosomes were depolarized with 33 mM KCl. The experimental data were expressed in nmol of glutamate released per mg of protein. The experiments were performed at 37 °C for 30–50 min with excitation wavelength of 360 nm and emission of 450 nm.

### Cannula Implantation

Mice were anesthetized with a mixture of ketamine (80 mg/Kg) and xylazine (10 mg/kg) injected intraperitoneally and placed in a stereotaxic apparatus. Small holes (0.9 mm) were drilled directed toward the CA1 region of dorsal hippocampus [dHIP: AP, −1.9; LL, ±1.6; DV, −1.0 ^36^] or main olfactory bulb [MOB: AP, +4.0; LL, ±1.0; DV, −3.0 ^27^]. Bilateral guide cannulae (22 G, 7 mm) containing dummy cannulae were inserted and fixed in the skull with zinc cement followed by dental acrylic. Coordinates were chosen based on Paxinos^37^. At least 7 days after, animals were used to pharmacological experiments.

### Electrode Implantation

Mice were anesthetized with a mixture of ketamine (80 mg/Kg) and xylazine (10 mg/kg) injected intraperitoneally and placed in a stereotaxic apparatus for implantation of three steel electrodes (0.005”, TeflonTM coated to a final thickness 0.007”, #7915, A-M SYSTEMS). One electrode was implanted in the dorsal HIP (AP −1.9; LL +1.6; DV −1.0), one in the ventral HIP (AP −2.8; LL +3.8; DV −1.0) and one in the mitral layer of the OB (AP +4.0; LL ±1.0; DV −3.0). One screw was used as reference and inserted in the contralateral occipital region, depth to dura mater; while a second one, used as ground screw, was inserted in the contralateral parietal region, also depth to dura mater. The electrodes and screws were positioned at specific depths and fixed to the skull with zinc cement. They were then welded to a pin connector, which allowed coupling to the electrophysiological recording system. At least 7 days after, animals were used to electrophysiology experiments.

### Pharmacological Experiments

At the time of the infusions, mice were gently held, dummy cannulas were removed and a cannula (30 G, 8 mm) was coupled to the guide cannula. Drugs were injected using a 10 μL syringe (HAMILTON, USA) connected to a pump adjusted to a flow of 0.5 μL/minute (min). After the end of microinjection, the syringe remained connected to the cannula for approximately 1 min to avoid reflux of the drug. All animals were gently restrained during the injection procedures. The experimental group received (0.5 μl/side) of NMDA (SIGMA, 1 μg/μL), AP5 (SIGMA, 1 μg/μL)^38^, AMPA (SIGMA, 1 μg/1 μL) or NBQX (SIGMA, 2 μg/μL)^39^. The control group received saline (0.5 μl/side).

### Social Recognition Test

Swiss juvenile male mice were used as intruders and were presented to the adult mouse inside a transparent acrylic cylinder (10 cm diameter) containing 60 holes equally distributed. Habituation phase consisted of introducing the adult mouse inside a clean standard cage containing an empty cylinder for 20 min. Training session (TR) lasted 5 min and consisted of replacing the empty cylinder by the one containing the juvenile mouse. Social investigation was quantified every time the resident animal introduced its nose and/or whiskers inside any of the cylinder’s holes. To assess social short-term memory (STM), 1:30 h after TR, mice were habituated exactly as describe above and the social investigation was scored. The social long term-memory (LTM) was measured 24 h after TR^40^. None of the juvenile mice were used more than three times.

### Electrophysiological recordings

A set of thin and light copper wires was developed for making it possible to proceed with the freely moving recordings. Local field potentials (LFP), recorded in the dorsal HIP and OB, were pre-amplified (gain of 2000), filtered (0.1 Hz high-pass filter and 500 Hz, low-pass filter) and digitized with a sampling frequency of 1 kHz (NATIONAL INSTRUMENTS). Data were visualized using the software KANANDA LTDA (Brazil) and recorded in a dedicated computer. Synchronized with the recording, live image of the animal was captured through a video camera (TVnPC P6).

### Electroencephalographic analysis (power spectrum and cross-frequency coupling)

All the analyses were performed using MATLAB R2013b, with codes available to the scientific community upon request. Sniffing segments were selected according to visual inspection of the video-EEG.

Spectral analysis was done using the short time Fourier transform (STFT), a time-frequency decomposition method, using windows with 1024 samples and 50% overlap. The energy of different LFP rhythms was extracted from each analysis segment (sniffing or no-sniffing), and mean values for each rhythm were taken for each recording and normalized by the respective baseline values (60 seconds before to juvenile exposition). Frequency bands were chosen as Delta 1–4 Hz; Theta 4–10 Hz; Alpha 10–16 Hz; Beta 17–30 Hz; slow Gamma 30–85 Hz and fast Gamma 90–140 Hz.

Functional connectivity between rhythms was assessed by estimating the degree of phase-amplitude coupling (PAC), a type of interaction which has received increasing interest because of its occurrence in several contexts, including the OB^41^. Furthermore, there are indications that this kind of coupling may play a key role in cognition and information processing^42–45^. PAC was measured between OB and dHIP using the Modulation Index (MI) proposed by Tort and colleagues^46^. The phase of the OB was obtained from the theta-filtered signal (4–10 Hz, the modulation band) and dHIP amplitude from the respective gamma-filtered signal (90–140 Hz), resulting in phase-amplitude distribution from which the MI was calculated. Thus, strong phase-amplitude modulation happens when gamma activity is concentrated in specific phases of the theta cycle, resulting in high MI values.

To account for the different exploration periods, MI was calculated in sliding windows of 1.8 seconds and 90% overlap. The mean values across windows of each sniffing segment were then obtained.

### Histology

The procedures for post-mortem verification of cannula and electrodes placements were made at the end of the behavioral tests, when animals were euthanized. The encephalon was removed and fixed with 4% PFA overnight, followed by placement in a 30% sucrose solution in PBS. The brains were maintained at 4 °C for 3 days. For the histological examination, 40 μm coronal brain slices were obtained using a cryostat. Only data from animals with the correct implantation of the guide cannula or electrodes were included in statistical analysis.

### Statistical analysis

Statistical analyses were performed using Prism 5 software (GRAPHPAD SOFTWARE). The data were analyzed by Two-way ANOVA and the post-hoc Bonferroni test for multiple comparisons. Unpaired t test was used to compare groups regarding glutamate release. Pearson correlation was performed between modulation index, theta or gamma power and recognition index. Significance level was set at p < 0.05.

## Results

### Region-specific effect of social isolation on glutamate release

**Figure 1:**
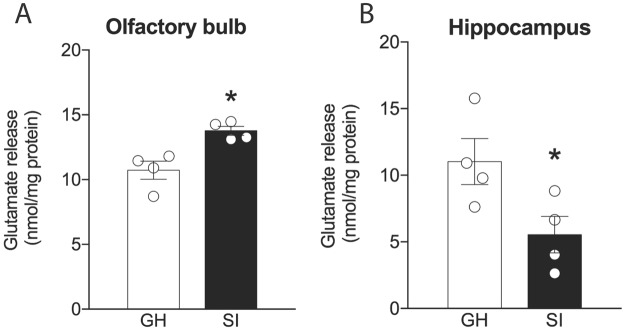
Effect of social isolation on the release of glutamate in the (**A**) olfactory bulb and (**B**) hippocampus. (n = 4/group). Results expressed as mean ± standard error of the mean. *Indicates difference between groups.

Glutamate signaling has been linked to the maintenance of hippocampus-dependent memories^47–49^. As socially isolated animals (SI) are unable to retain social memory for 24 h^16,18^, we tested whether SI would compromise neuronal excitability by altering the glutamate release. We prepared synaptosomes from olfactory bulb (OB) and hippocampus (HIP) of naïve group-housed (GH) and isolated (SI) mice. High potassium stimulated synaptosomes from the OB of SI mice released more glutamate compared to group-housed (GH) animals [t_(6)_ = 3.9, p = 0.007; Fig. 1A]. On the contrary, glutamate release decreased in hippocampal synaptosomes from SI mice [t_(7)_ = 2.5, p = 0.04; Fig. 1B]. Thus, our results suggest that SI increases the glutamatergic tonus in the OB, while decreases in the HIP.

### Blocking AMPA and NMDA receptors into the OB allows social memory persistence in SI mice

**Figure 2:**
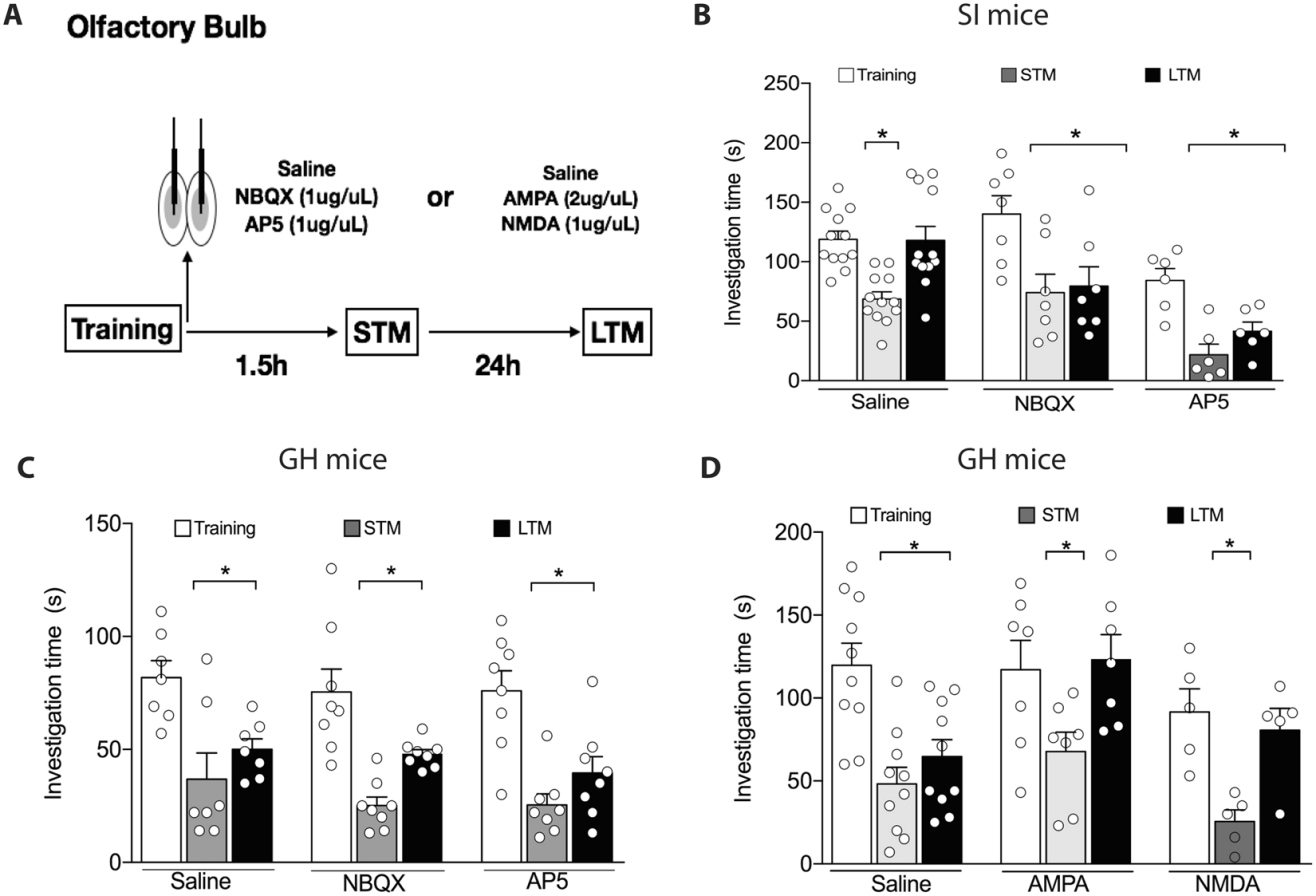
Effect of NBQX (AMPA receptor antagonist) and AP5 (NMDA receptor antagonist) administration in the olfactory bulb (OB), immediately after the training session on the social recognition task. (**A**) Schematic representation of the experimental design. (**B**) Blockade of AMPA (NBQX) and NMDA (AP5) receptors into the OB recovered the social long-term memory (LTM) in social isolated (SI) mice without affecting social short-term memory (STM) (n = 6–12/group). (**C**) NBQX and AP5 into the OB did not affect STM and LTM in group-housed (GH) mice (n = 7–8/group). (**D**) AMPA and NMDA agonists impaired LTM in GH animals (n = 5–10/group). Results are expressed as mean ± standard error of the mean. *Indicates difference between the training and test sessions, within the same group.

Next, we tested whether the higher levels of glutamate release into the OB were driving the memory deficits in SI mice, by blocking AMPA (NBQX) and NMDA (AP5) receptors directly into the OB (Fig. 2A). As we predicted, the intra-OB injection (immediately after the TR) of NBQX and AP5 rescued LTM (tested 24 h after training) in SI mice, without affecting STM (tested 1 h 30 min after training) [Interaction: F_(4,46)_ = 3.6, p = 0.01; Drug: F_(2,23)_ = 7.3, p = 0.003; Trial: F_(2,46)_ = 37.5, p < 0.0001; Fig. 2B].

To test whether glutamatergic signaling in the OB is necessary to social memory consolidation, we administered the same antagonists, NBQX and AP5, in group-housed animals (GH). Our results showed that AMPA and NMDA activation in the OB is not necessary to social memory consolidation [Interaction: F_(4,40)_ = 0.2, p = 0.9; Drug: F_(2,20)_ = 0.9, p = 0.3; Trial: F_(2,40)_ = 39.4, p < 0.0001; Fig. 2C].

Next, we designed a pharmacological experiment to reinforce the cause-effect relationship between high glutamate levels into the OB and social memory persistence. AMPA and NMDA agonists were administered directly into the OB of GH animals, immediately after training. Our results showed that AMPA and NMDA [Interaction: F_(4,38)_ = 2.6, p = 0.05; Drug: F_(2,19)_ = 3.3, p = 0.05; Trial: F_(2,38)_ = 23.6, p < 0.0001; Fig. 2D] impaired LTM, but not STM, suggesting that increasing glutamatergic tonus into the OB, either by AMPA and NMDA agonists compromised social memory persistence.

### Activating AMPA receptors in the dorsal hippocampus (dHIP) allows social memory persistence in SI mice

**Figure 3:**
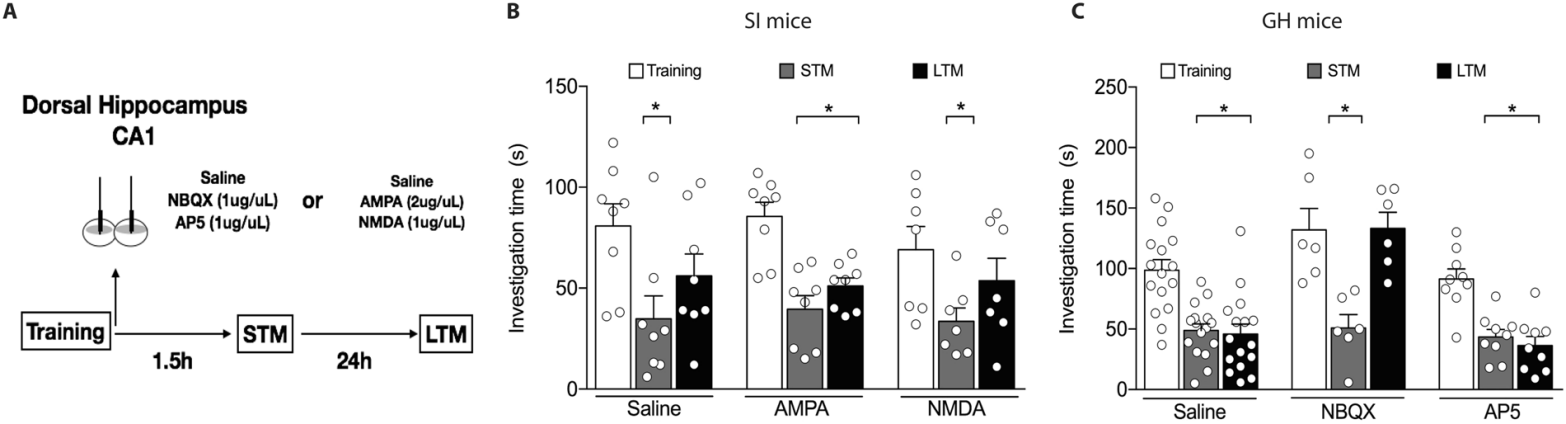
Effect of AMPA and NMDA receptors agonists administration in the dorsal hippocampus (dHIP), immediately after the training session on the social recognition task. (**A**) Schematic representation of the experimental design. (**B**) AMPA agonist into the dHIP recovered the social long-term memory (LTM) deficit of social isolated mice (SI). NMDA agonist did not recover LTM (n = 7–8/group) of SI mice. (**C**) Administration of NBQX impairs the consolidation of LTM in group-housed animals (GH), while, the administration of AP5 did not affect LTM (n = 6–16/group). Results are expressed as mean ± standard error of the mean. *Indicates difference between the training and test sessions, within the same group.

The activation of glutamatergic receptors into the hippocampus seems essential for the consolidation of LTM^50,51^. However, fear conditioning was the behavioral paradigm used to demonstrate this dependency. Thus, before testing whether decreased glutamate release from hippocampal synaptosomes would have a cause-effect relationship with LTM impairment, we tested the hypothesis that hippocampal glutamatergic signaling is essential for the formation of long-lasting social memory. To address this question, AMPA and NMDA receptors antagonists were administered into the CA1 region of the dHIP, immediately after TR in the social recognition test (Fig. 3A).

NBQX impaired the consolidation of LTM in GH animals, though AP5 had no effect [Interaction: F_(4,56)_ = 7.6, p < 0.0001; Drug: F_(2,28)_ = 10.5, p = 0.0004; Trial: F_(2,56)_ = 39.1, p < 0.0001], Fig. 3C.

After, we verified whether the activation of AMPA and NMDA receptors in the hippocampus of isolated animals would restore LTM in these animals. Intra-hippocampal AMPA activation recovered LTM in SI mice [Interaction: F_(4,40)_ = 0.49, p = 0.74; Drug: F_(2,20)_ = 0.22, p = 0.8; Trial: F_(2,40)_ = 25.93, p < 0.0001], while NMDA had no effect (Fig. 3B).

Altogether, our results suggest that the hippocampal activation of AMPA receptors, rather than NMDA receptors, in the early stages of consolidation is necessary for the formation of LTM.

### Social exploration increases gamma, but not theta power into the OB and dHIP

**Figure 4:**
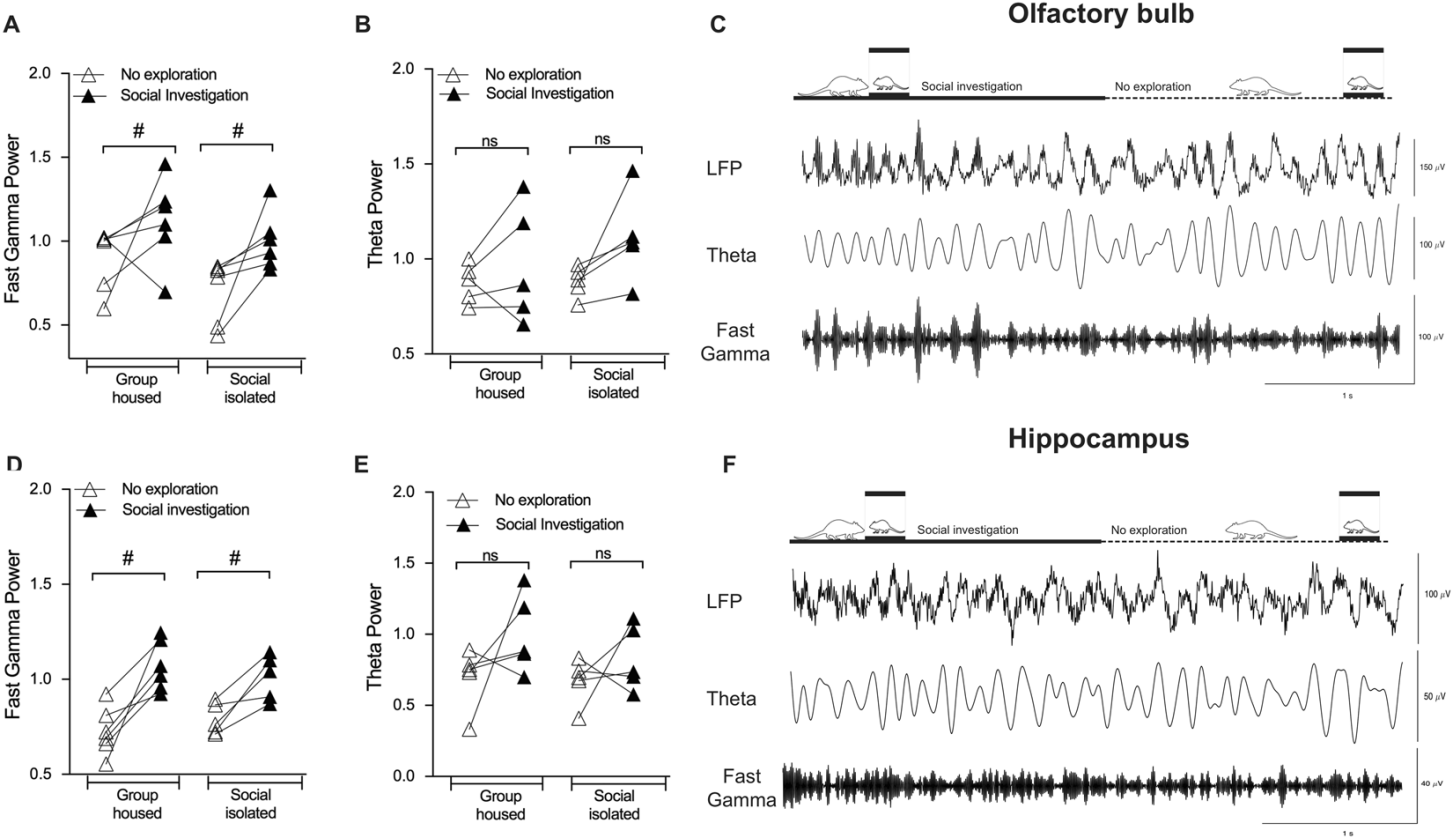
Effect of social exploration on fast gamma and theta oscillations in the (**C**) olfactory bulb (OB) and (**F**) dorsal hippocampus (dHIP). Social exploration increases fast gamma power in the (**A**) OB (n = 6/group) and (**D**) dHIP (n = 5/group), in both groups. ^#^Indicates difference between no exploration and social exploration within the same group. There was no difference in theta power after social exploration in (**B**) OB (n = 6/group) and (**E**) dHIP (n = 5/group) of both groups.

Glutamatergic signaling may drive gamma oscillations in the OB^41^ reviewed by^33^ and theta oscillations in the HIP^52^. Furthermore, in humans, it was showed a robust relationship between hippocampal glutamate levels and frontal theta activity^53^. As we showed evidences that SI changed glutamatergic transmission in the OB and HIP, compromising social memory persistence. For that purpose, we recorded the local field potential (LFP) in both areas, during the social recognition test. First, we verified whether social exploration would change brain activity in both groups. We observed that social investigation increased the fast gamma power into the OB of both GH and SI animals [Interaction: F_(1,18)_ = 0.06, p = 0.8; Environment condition: F_(1,18)_ = 6.0, p = 0.02; Behavior: F_(1,18)_ = 16.6, p = 0.0007; Fig. 4A,C]. No effect on theta power was observed [Interaction: F_(1,16)_ = 0.5, p = 0.4; Environment condition: F_(1,16)_ = 0.6, p = 0.4; Behavior: F_(1,16)_ = 3.1, p = 0.09; Fig. 4B,C].

In the HIP, social investigation increased fast gamma power in both groups [Interaction: F_(1,18)_ = 1.4, p = 0.2; Environment condition: F_(1,18)_ = 0.005, p = 0.9; Behavior: F_(1,18)_ = 31.0, p < 0.0001; Fig. 4D,F]. Similar to OB, no effect of social exploration in hippocampal theta power was detected in GH and SI mice [Interaction: F_(1,16)_ = 0.5, p = 0.4; Environment condition: F_(1,16)_ = 0.9, p = 0.3; Behavior: F_(1,16)_ = 5.4, p = 0.03; Fig. 4E,F].

Considering these results, we suggest that social exploration triggers fast gamma oscillations in the OB, regardless of whether the animal was submitted or not to social isolation.

### Social isolation increased gamma power oscillations in the olfactory bulb, during long-term social memory retrieval

**Figure 5:**
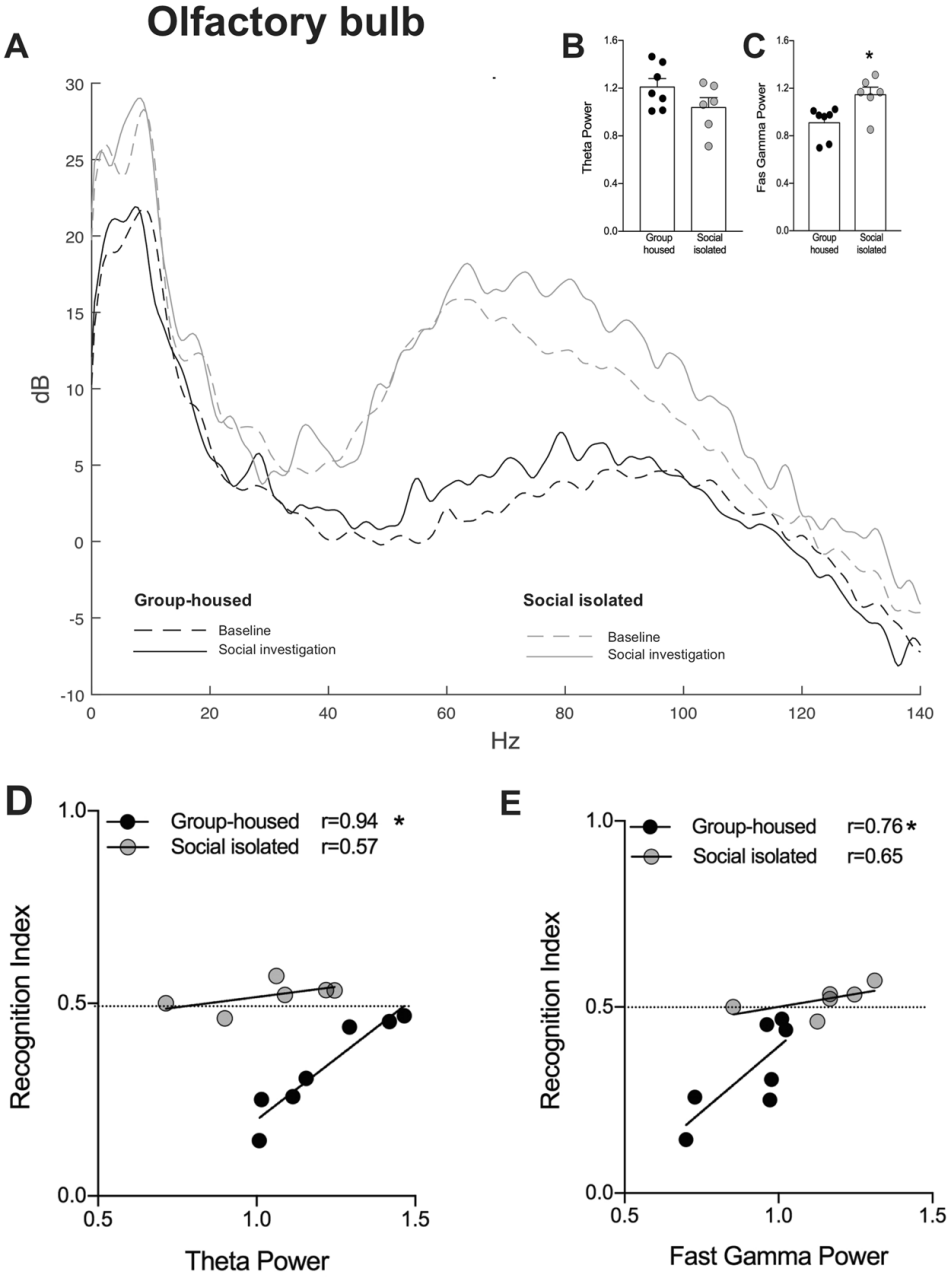
Effect of social isolation on the olfactory bulb gamma and theta oscillations, during social long-term memory retrieval (LTM). (**A**) Power Spectral Density (PSD) from one representative animal from group-housed (dark lines) and social isolated (grey lines) groups. (**B**) Theta and (**C**) fast gamma power during LTM. *Indicates difference between groups. Correlation between LTM performance (recognition index) and (**D**) theta or (**E**) fast gamma power oscillation. *Indicates statistically significant correlation.

Next, we evaluated the OB oscillations during memory retrieval. No difference between groups was observed in STM (data not shown). Figure 5A shows the power spectral density (PSD) of LFP recordings in the OB of one animal from each group. We did not observe difference between group-housed and isolated mice for theta oscillations (t_(11)_ = 1.6, p = 0.13; Fig. 5B). However, social isolation increased fast gamma oscillations during LTM retrieval (t_(11)_ = 2.8, p = 0.01; Fig. 5C).

In our study, changes in theta and gamma power may not be related to speed of locomotion^54,55^, since no difference on speed of locomotion between GH and SI mice was detected (Fig. S1).

Next, we asked whether OB theta and gamma oscillations were driving the social memory retrieval in both conditions: group-housed and social isolation. Theta and gamma power linearly correlated with memory performance (recognition index) in group-housed animals (Theta: r = 0.94, p = 0.001; Fast gamma: r = 0.76, p = 0.04), but not in SI mice (Theta: r = 0.57, p = 0.23; Fast gamma: r = 0.65, p = 0.15), Fig. 5D,E.

### Social isolation did not alter gamma and theta power oscillations in the dorsal hippocampus, during long-term social memory retrieval

**Figure 6:**
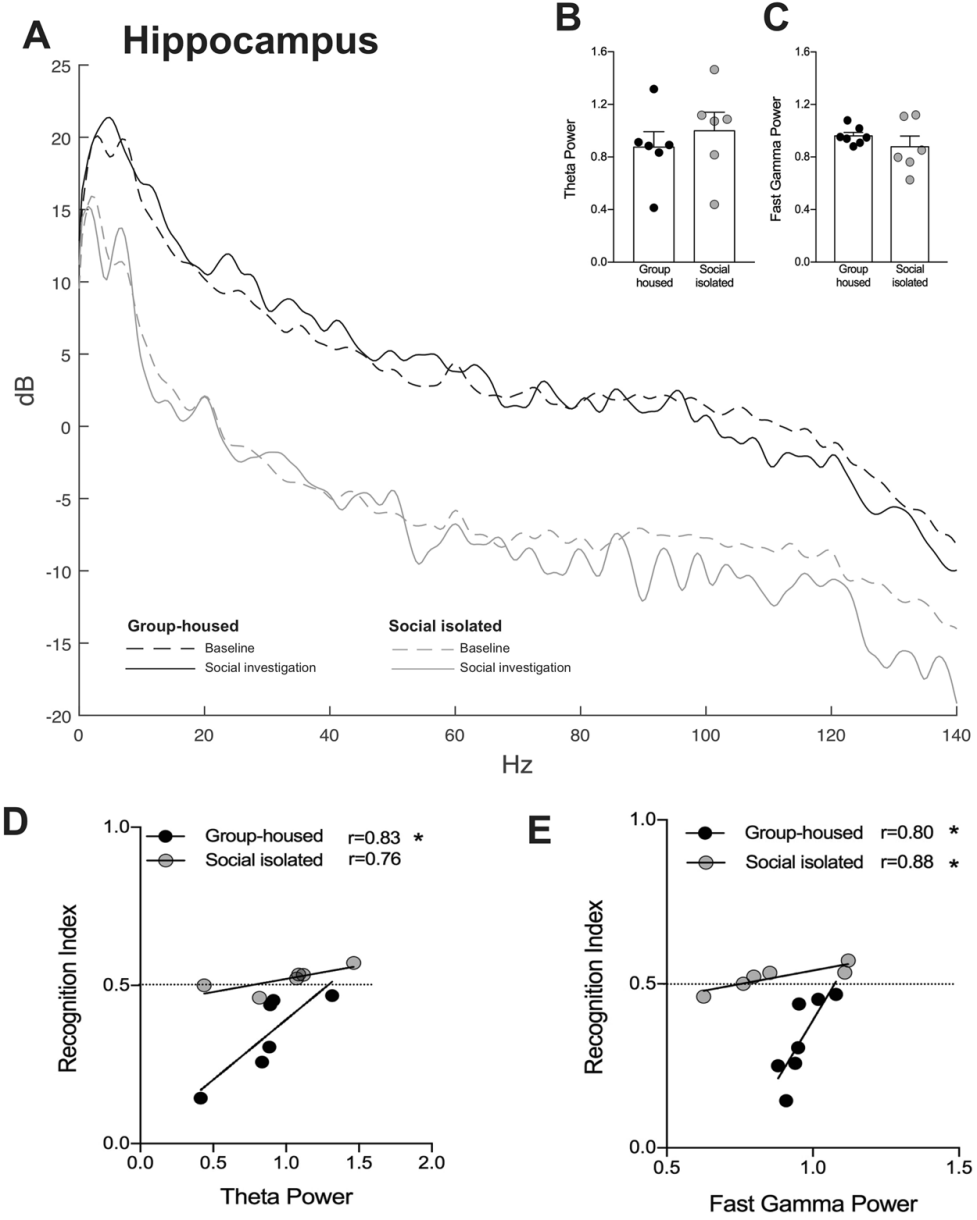
Effect of social isolation on the dorsal hippocampus fast gamma and theta oscillations, during social long-term memory retrieval (LTM). (**A**) Power Spectral Density (PSD) from one representative animal from group-housed (dark lines) and social isolated (grey lines) groups. (**B**) Theta and (**C**) fast gamma power during LTM. Correlation between LTM performance (recognition index) and (**D**) theta or **(E**) gamma power oscillation. *Indicates statistically significant correlation.

To further explore the effect of SI on hippocampal oscillations, we quantified the fast gamma and theta power during the memory retrieval. No differences between groups were observed during STM (data not shown). Figure 6A shows the power spectral density (PSD) of LFP recordings in the dorsal hippocampus (dHIP) of one animal from each group. We did not observe statistical difference between groups regarding theta (t_(10)_ = 0.67, p = 0.51; Fig. 6B) and fast gamma (t_(11)_ = 1.0, p = 0.31; Fig. 6C) power oscillations.

Next, we evaluated whether there is correlation between LTM and hippocampal oscillations, and whether social isolation plays a role in this correlation. There was a significant correlation between social memory and theta oscillations in group-housed mice (r = 0.83, p = 0.03; Fig. 6D), but not in SI mice (r = 0.76, p = 0.07; Fig. 6D). However, both groups (Group-housed: r = 0.80, p = 0.02; SI: r = 0.88, p = 0.01; Fig. 6E) showed significant correlation between LTM performance and dHIP fast gamma oscillations. The correlation analysis of group-housed animals indicates that social memory is evoked more efficiently the lower the value for dHIP gamma and theta power.

### Social isolation decreased theta phase/gamma amplitude coupling between the OB and the dorsal HIP during long-term social memory retrieval

LFP analysis indicated that gamma and theta power oscillations, in both OB and dHIP, correlates with animal’s performance during LTM retrieval, in GH animals. However, on average, we only observed a difference between GH and SI animals in OB gamma power. Therefore, these results, though interesting, did not entirely explain why SI mice do not present LTM.

**Figure 7:**
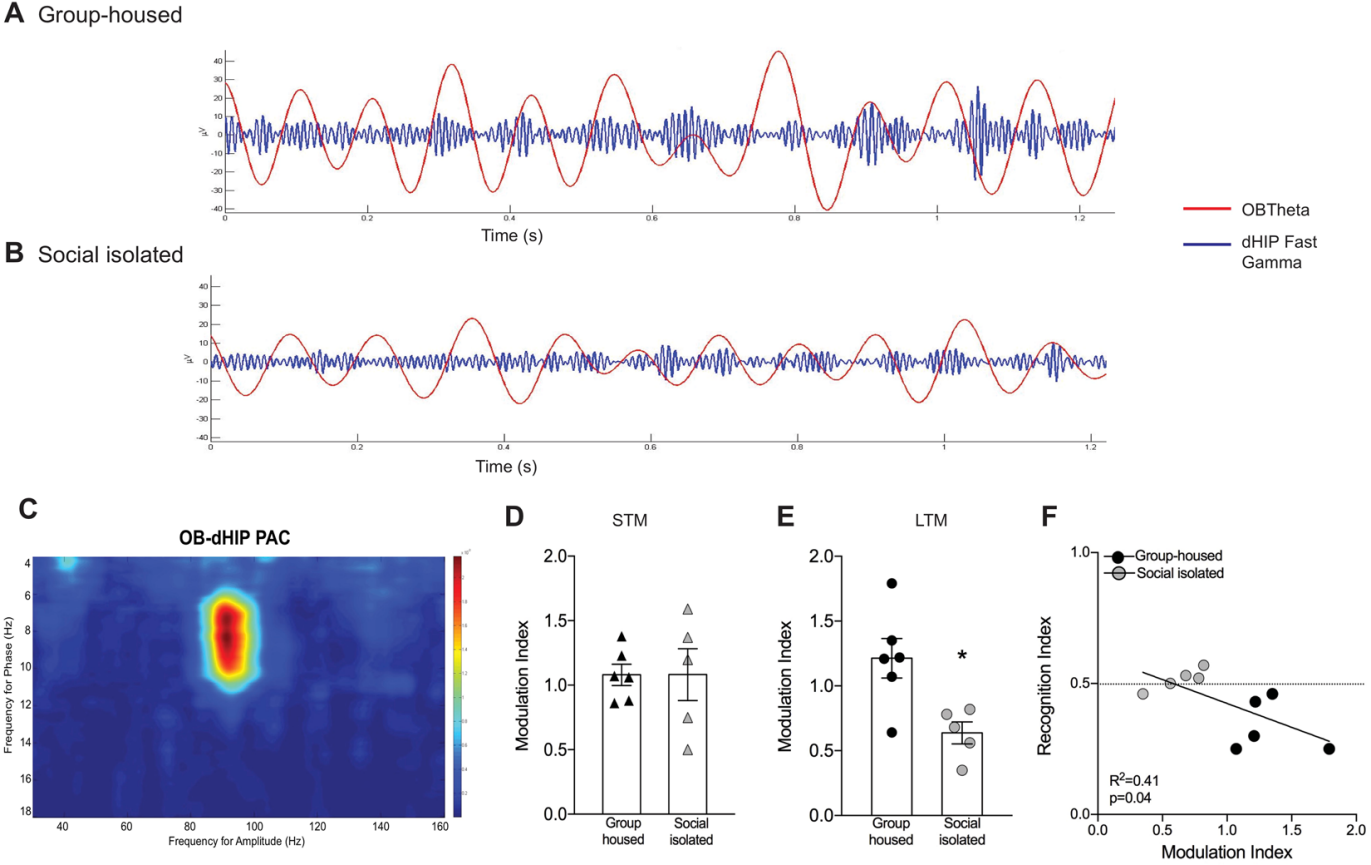
Theta phase/gamma amplitude coupling between the olfactory bulb (OB) and dorsal hippocampus (dHIP) during the retrieval of short (STM) and long-term memory (LTM). Representative theta phase/gamma amplitude coupling between the OB and dHIP during LTM in (**A**) group-housed and (**B**) isolated animal. (**C**) Representative phase-amplitude comodulogram recorded during social exploration. Modulation index during (**D**) STM and (**E**) LTM. *Indicate difference between groups. (**F**) Positive correlation between LTM performance (recognition index) and coupling (modulation index).

Thus, we decided to evaluate OB-dHIP circuits by analyzing the strength of coupling between LPF rhythms: theta phase and fast gamma amplitude (Fig. 7A,B). We did not find coupling between OB and ventral hippocampus and between both hippocampus (data not shown). The functional connectivity, measured by the interactions among frequency bands, revealed that OB modulates dHIP during STM (Fig. 7D) and LTM (Fig. 7E) in GH and SI animals. However, SI animals showed a lower coupling during LTM [t_(9)_ = 3.0, p = 0.01], while were similar to GH during STM retrieval [t_(9)_ = 0.0005, p = 0.3].

To further characterize the effect of OB-dHIP coupling and LTM, we also analyzed the correlation between coupling (modulation index) and performance in LTM (recognition index). Correlation analysis shows that recognition index correlated with modulation index [r = −0.64; p = 0.04; Fig. 7F]. Also, the linear regression indicated that efficient coupling (high values of modulation index) was present in animals showing LTM (see black circles, GH animals, Fig. 7F). Our results indicated a significant coefficient of determination (R^2^), p = 0.04, with explanatory value for 53% of all cases (R^2^ = 0.41).

## Discussion

The present study shows that social isolation (SI) potentiates the glutamatergic signaling in the OB, while reduces in the HIP. The glutamatergic unbalance lead to a deficit in LTM, which is accompanied by a decreased phase-amplitude coupling between dHIP and OB.

We found increased glutamate release from OB synaptosome after social isolation. In addition, blocking AMPA and NMDA receptors in the OB, after training, rescued social memory in SI mice, while did not change in GH animals. In line with these results, AMPA and NMDA activation mimicked the SI impairment in control animals. Regardless of whether the source of glutamate has not been accessed, increased glutamatergic signaling in the OB was deleterious to LTM. However, we wonder how would SI be changing the glutamatergic tonus in the OB?

Depriving neonatal rats of odors, through naris occlusion, during 3 days, increased the probability of glutamate release in the glomerulus, and also increased synaptic currents mediated by AMPA and NMDA receptors^24^. Thus, one possibility is that under SI, the animal is subjected to a restricted repertoire of odors, diminishing the sensorial input to the OB and leading to an upregulation of the glutamatergic system. Accordingly, we showed previously that if the SI is applied in combination with odors from conspecific, the LTM deficit is rescued^18^.

Nevertheless, the effects of social isolation go beyond the olfactory constraint. Mice rely on social contact to maintain mental and physical health^56–58^. One of the main mediators of sociability is the neuropeptide oxytocin (OXT). OXT administration usually increases pro-social behaviors in several species^59–61^. Furthermore, engaging in social interactions may trigger the production of OXT^62,63^.

Recently, it was found that OXT, acting on the anterior olfactory nucleus (AON), adjusts the feedback inhibition of mitral cells, improving the signal-to-noise ratio of odor responses^64^. Additionally, same authors found that deletion of OXT receptors in the AON increased olfactory exploration and impaired specifically the social recognition memory^64^. Accordingly, the activation of the par medialis portion of AON worsened social recognition^65^. Collectively, these studies reinforce the idea that centrifugal feedback excitatory projections to the OB are important to adjust olfactory sensitivity^66^ and, further, suggest that OXT may play a role in this process. We, therefore, speculate that SI may affect oxytocin production and/or signaling, ultimately leading to a glutamatergic unbalance in the OB. As a consequence, the signal-to-noise ratio for juvenile’s odors detection in mitral/tufted cells is compromised, as well as the social recognition memory.

Feedback inhibition of mitral cells firing by granule cells has been proposed as the possible generator of OB gamma oscillations^22^. Accordingly, odor stimulation, as well as olfactory learning, modify gamma oscillation in the OB^67^. During the TR, the adult mouse samples the juvenile, mainly using its olfactory system, likely reason why gamma power increased in OB, in response to the social investigation. At this point, social isolation had no effect, since no difference between groups was observed, suggesting that SI is affecting the storage of social memory rather than olfaction *per se*.

It has been reported that social recognition memory is a hippocampus-dependent memory^16,28,68^. Here we demonstrated that AMPA, but not NMDA receptors activation in the CA1 region of the dHIP is necessary to social recognition memory, reinforcing the idea of the hippocampus recruitment in this type of memory. Furthermore, SI decreased glutamate release from hippocampal synaptosomes. As we recovered the memory deficit of SI mice by the intra-hippocampal administration of AMPA, but not NMDA, we strongly suggest a cause-effect relationship between glutamatergic signaling and LTM consolidation in the dHIP. In line with these observations, extensive research has demonstrated that AMPA administration into the hippocampus improves memory^69–72^. Notably, odor discrimination learning is facilitated following injection of AMPA agonist^73^.

We argued before that OXT^64^ could be a candidate to participate on the effect of SI on the glutamatergic signaling in the OB. Thus, we wonder whether a similar mechanism could be proposed to explain the effects of SI in the hippocampus. Intranasal OXT increases both gamma power and activation, measured by functional magnetic resonance, of the dHIP^74^. It was recently demonstrated that hippocampal OXT receptors are necessary for discrimination of social stimuli^75^. Same authors identified that hippocampal OXT receptors are preferentially expressed in inhibitory interneurons, which reconcile with results showing that OXT receptors activation disinhibits CA1 neurons^76^. Therefore, in our study, we suggest that SI could be interfering in the oxytocin ability to modulate hippocampus excitability, compromising LTM.

Social memory can be studied using distinct paradigms. In the habituation-dishabituation protocol, memory is formed by short and repeated exposition to a conspecific, taking to the formation of a short-term memory trace^77,78^. It was shown that theta oscillations, in the main OB of rats, gradually decrease along the encounters in the habituation-dishabituation protocol, indicating a strong correlation between olfactory exploration and theta rhythms^79^. In our study, we did not observe a relation between OB or dHIP theta oscillations and social exploration, during the acquisition phase. However, during LTM retrieval, we observed a significant correlation in control animals, whereas animals with better performance were the ones with lower theta power in the OB and dHIP.

Theta oscillations is frequently associated with communication between distant areas^80^ and linked with cognitive performance^79^. Furthermore, simple home cage exploration may induce theta oscillations^81^. Alternatively, it has been argued that theta oscillations in areas such as dHIP and OB may be in fact a respiration-entrained rhythm [RR^82–86^]. We did not measure nasal respiration, though the quantification of social investigation resembles sniffing. Therefore, the theta power quantified here could be overestimated and be in fact a RR. If this was true, we should have seen higher theta power when animals were exploring the juvenile, such as in the training session. However, we did not find an increase in theta power in both OB and dHIP when animals were exploring the juvenile.

Finally, we tested the hypothesis that SI impaired LTM by compromising the communication between OB and dHIP. To test this hypothesis, we chose to analyze the phase-amplitude coupling, or cross-frequency coupling (CFC) between OB and dHIP and represent this result as modulation index [MI^46^]. Phase-amplitude coupling may be useful to assess the communication between brain areas, whereas slow oscillation phase (theta) modulates the amplitude of faster oscillation (gamma)^43^. Several studies have shown that theta-gamma coupling may facilitate transfer of information throughout entorhinal cortex-hippocampus network^87^. And recently, we found OB-dHIP CFC during a spatial olfactory task^88^.

We observed a theta phase-gamma amplitude coupling between OB and dHIP during social memory retrieval in GH animals. The involvement of an olfaction-hippocampal network has been shown in odor processing and discrimination^33,89,90^. Here, we indicate that during social memory retrieval, gamma oscillations in the dHIP are driven by the OB. Accordingly, a potential function for higher amplitudes of gamma oscillations at a particular theta phase is to facilitate memory recollection of earlier experiences^91–94^.

As predicted, MI during LTM, but not STM, was attenuated by SI. We also found that MI can predict the LTM performance, with explanatory value of 53%. There are several studies showing that impairment in hippocampal gamma amplitude modulation by theta phase relates to memory deficits^95,96^. However, this is the first time a study shows OB-dHIP coupling in social recognition memory.

Social isolation impaired the glutamatergic transmission in the OB and dHIP at the same time that decreased the theta-phase gamma-amplitude coupling between same areas, during LTM retrieval. In the present study we did not address whether the pharmacological manipulations of AMPA and NMDA receptors, performed immediately after training, would affect OB-dHIP coupling at the time that LTM is tested. However, there are some evidences showing that drugs that alter the glutamatergic tonus, in areas such as dorsal hippocampus^97^ and the olfactory bulb^98^, modified brain oscillations and related-behaviors.

Altogether, our results show that SI induced opposite glutamatergic tonus in OB and dHIP, which is one possible cause for the social memory deficit. Furthermore, we may suggest that disturbing glutamatergic signaling compromised the normal communication between OB and HIP, impairing social memory persistence.

## Supplementary information

Supplementary information
