# Supplementary material for: Social isolation impairs the persistence of social recognition memory by disturbing the glutamatergic tonus and the olfactory bulb-dorsal hippocampus coupling
Source: Sci Rep. 2019 Jan 24;9:473. doi: 10.1038/s41598-018-36871-6 (PMC6345767; doi:10.1038/s41598-018-36871-6)
Supplement: Supplementary file 1 — Supplementary information [file 41598_2018_36871_MOESM1_ESM.docx]

**Social isolation impairs the persistence of social recognition memory by disturbing the glutamatergic tonus and the olfactory bulb-dorsal hippocampus coupling**

Ana F. Almeida-Santos^1^, Vinícius R. Carvalho^2^, Laura Jaimes^1^, Caio M. de Castro^1^, Hyorrana P. Pinto^1^, Tadeu P. D. Oliveira^3^, Luciene B. Vieira^3^, Márcio F. D. Moraes^1^, Grace S. Pereira^1*^.

^1^Núcleo de Neurociências, Departamento de Fisiologia e Biofísica, Instituto de Ciências Biológicas, Universidade Federal de Minas Gerais, Brazil.

^2^Departamento de Engenharia Eletrônica, Escola de Engenharia, Universidade Federal de Minas Gerais, Brazil.

^3^Laboratório de Neurofarmacologia, Departamento de Farmacologia, Instituto de Ciências Biológicas, Universidade Federal de Minas Gerais, Brazil.

*Correspondence and requests for materials should be addressed to G.S.P.

grace@icb.ufmg.br.


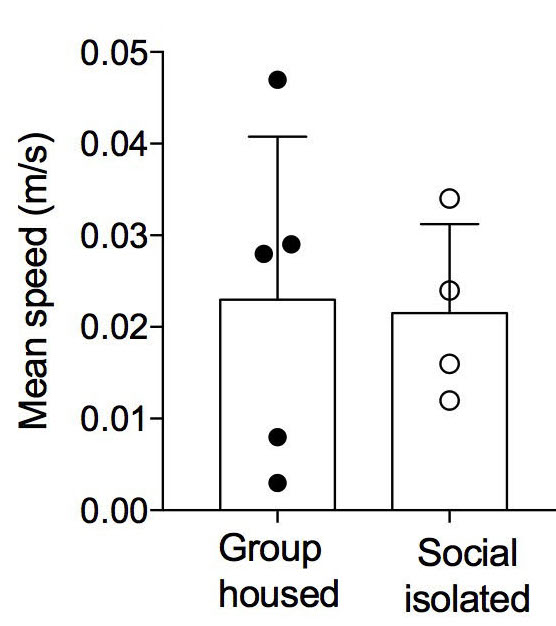
**Supplementary Figure S1.** Speed of locomotion during the long-term memory test (LTM) measured by Any-Maze^®^ (Stoelting, IL, USA; version 4.5) software. Data are represented as mean±SEM. There is no difference between groups [unpaired t-test: t_(7)_= 0.1, p= 0.8].

**Supplementary Figure S2.** Theta phase/ slow gamma amplitude coupling between the olfactory bulb (OB) and dorsal hippocampus (dHIP) during the retrieval of short (STM) and long-term memory (LTM). Modulation index during (A) STM [unpaired t-test: t(10)= 1.9, p= 0.08] and (B) LTM [unpaired t-test: t(9)= 1.1, p= 0.2].


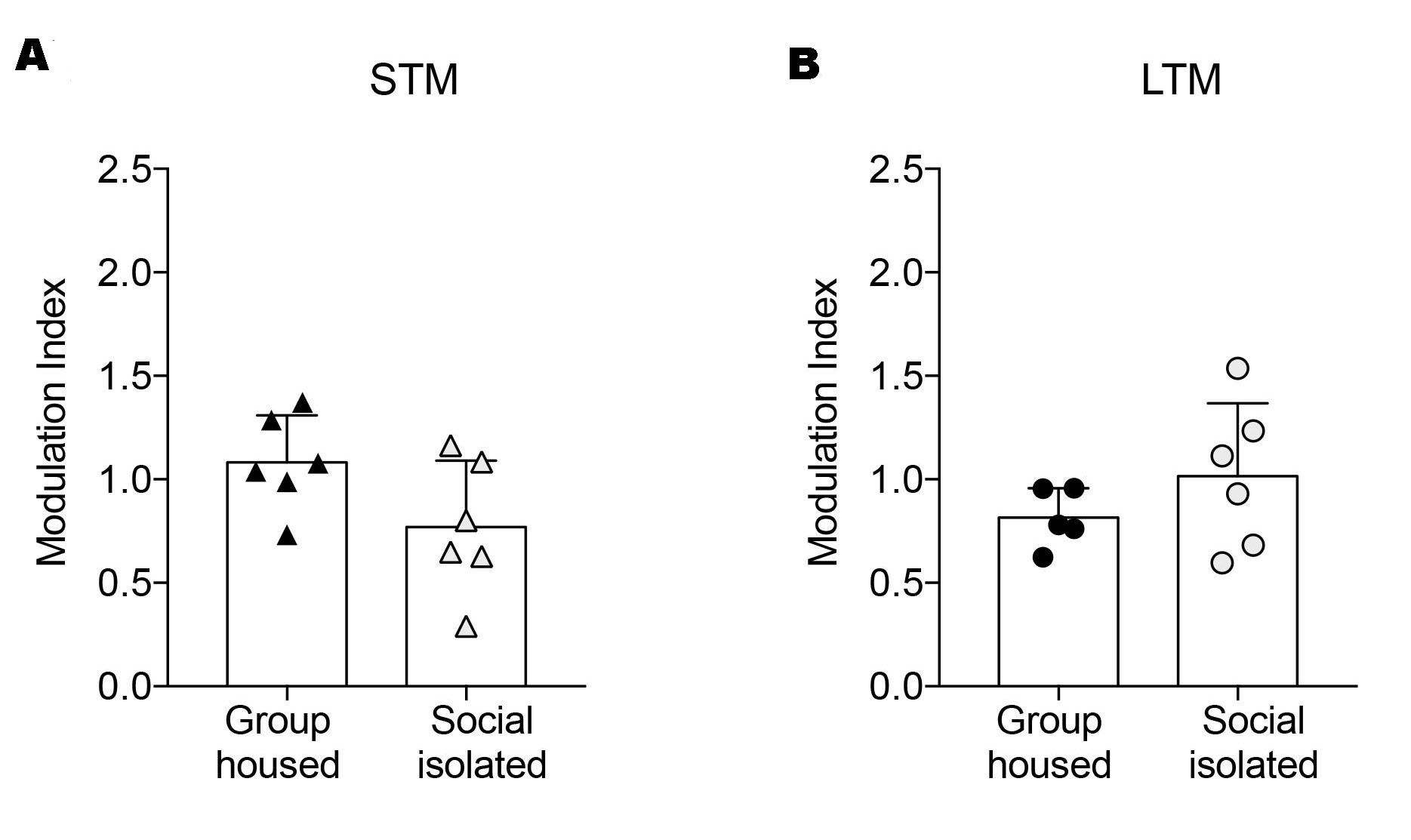


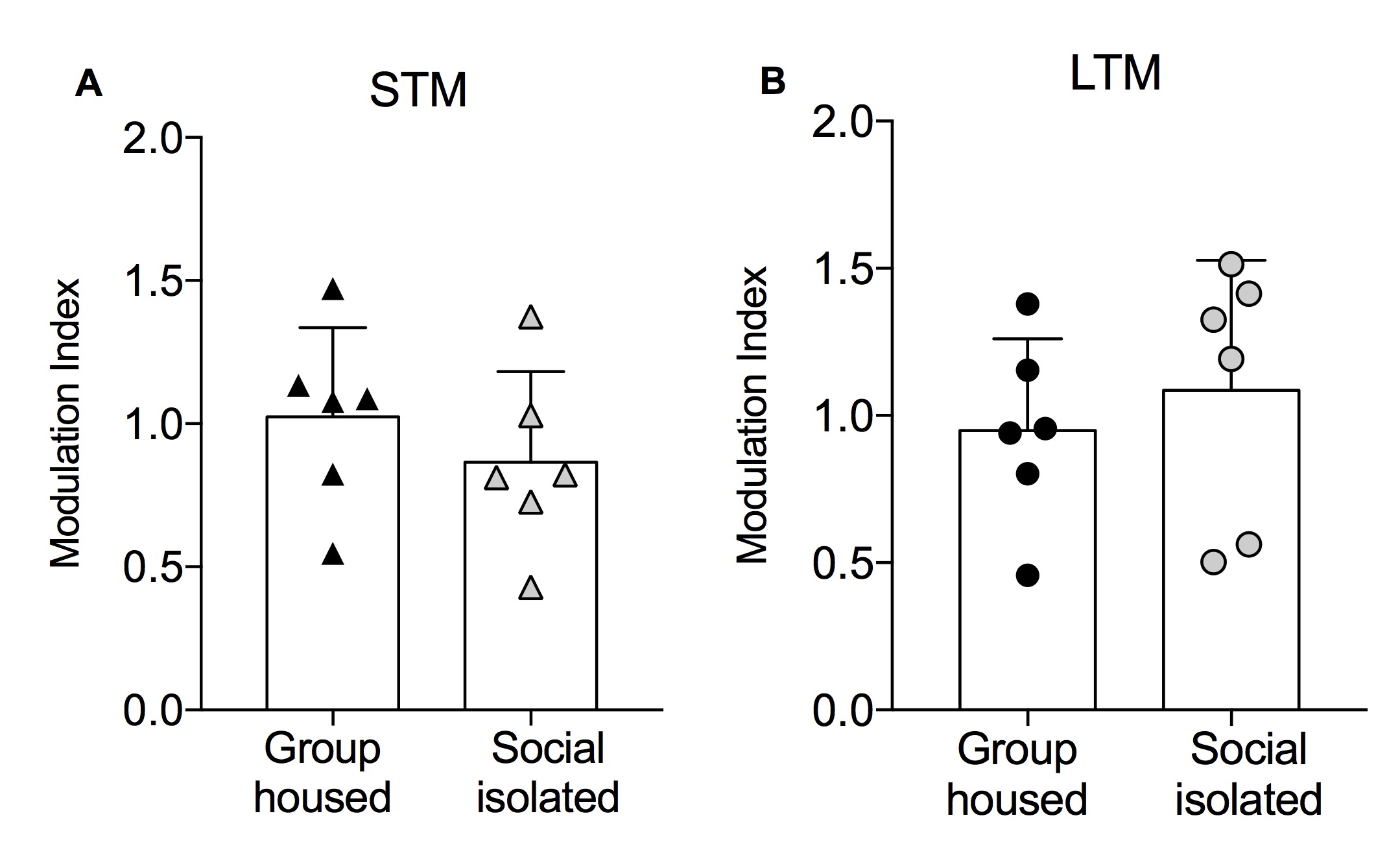
**Supplementary Figure S3.** Theta phase/ fast gamma amplitude coupling within the dorsal hippocampus (dHIP) during the retrieval of short (STM) and long-term memory (LTM). Modulation index during (A) STM [unpaired t-test: t(10)= 0.8, p= 0.4] and (B) LTM [unpaired t-test: t(10)= 0.6, p= 0.5].

**Supplementary Figure S4.** Theta phase/ fast gamma amplitude coupling between the olfactory bulb (OB) and dorsal hippocampus (dHIP) during the training phase of the social recognition test [unpaired t-test: t(9)= 1.6, p= 0.1].

**Supplementary Figure S5.** Theta phase/ fast gamma amplitude coupling between the olfactory bulb (OB) and dorsal hippocampus (dHIP) in the three phases of social recognition memory: training, short-term (STM) and long-term memory test (LTM). Modulation index of (A) Group housed [Repeated one-way ANOVA with Dunnett’s post-hoc: F_(2,10)_= 1.5, p= 0.2] and (B) social isolated mice [Repeated one-way ANOVA with Dunnett’s post-hoc: F_(2,8)_= 3.9, p= 0.1].
